# Role of multidimensional evaluations in the support of school trajectories of children with mild to moderate intellectual disability

**DOI:** 10.1192/j.eurpsy.2021.352

**Published:** 2021-08-13

**Authors:** N. Touil, A. Curie, M.-P. Reymond, F. Subtil, S. Roche, S. Gaillard, B. Kassai, V. Des Portes

**Affiliations:** 1 Filière Défiscience, HOSPICES CIVILS DE LYON, Bron, France; 2 Service De Neurologie Pédiatrique, HOSPICES CIVILS DE LYON, Bron, France; 3 Pôle De Santé Publique, Service De Biostatistiques, HOSPICES CIVILS DE LYON, BRON, France; 4 Service De Biostatistiques, HOSPICES CIVILS DE LYON, Bron, France; 5 Epicime-cic 1407 De Lyon, HOSPICES CIVILS DE LYON, BRON, France

**Keywords:** multidimensional evaluations, psychometric evaluation, Intellectual disability

## Abstract

**Introduction:**

There is a lack of objective evaluation with validated tools in school children with intellectual disability (ID). Standardized and validated tools, allowing children evaluations and follow-up, exist but are poorly used. Our action-study wishes to develop evaluation practices to better adapt to the specific needs of children with ID.

**Objectives:**

We evaluated the multidimensional profiles (cognitive, adaptative and behavioral) of children with ID attending regular or adapted school system.

**Methods:**

School children, aged 5 to 13 years old, with mild to moderate ID were enrolled in this French cohort study. The multidimensional evaluation consisted of a school evaluation grid proposed by the French educational system, a scale of school needs (GEVA-sco), an intellectual assessment (WISC IV), a behavior adaptative scale (Vineland II) and a behavior rating scale (the French Nisonger Child Behavior Rating Form (Nisonger CBRF)). The results of this multidimensional assessment were analyzed.

**Results:**

Between November 2014 and June 2016, 121 children were enrolled, 3 children were lost to follow-up. Analysis was performed on 118 children. Seventy one (60.2 %) were male. Fifty-two (44.1%) were aged 6 to 9 years. Sixty-eight (57.6%) children were in regular schools and 50 (42.4%) in adapted schools. Children in regular schools had a higher mean IQ score (57.5) than children in adapted schools (43.5). The adaptative behavior profile of children in regular school is less severe than in children in adapted schools.

**Conclusions:**

Multidimensional evaluations allow optimizing and personalizing support. Evaluation of adaptative behavior is more informative than cognitive profile which does not differentiate between children skills

**Disclosure:**

No significant relationships.

